# Gut microbiota is involved in male reproductive function: a review

**DOI:** 10.3389/fmicb.2024.1371667

**Published:** 2024-05-03

**Authors:** Shuya Lv, Jingrong Huang, Yadan Luo, Yuhang Wen, Baoting Chen, Hao Qiu, Huanxin Chen, Tianhao Yue, Lvqin He, Baochun Feng, Zehui Yu, Mingde Zhao, Qian Yang, Manli He, Wudian Xiao, Xiaoxia Zou, Congwei Gu, Ruilin Lu

**Affiliations:** ^1^Laboratory Animal Centre, Southwest Medical University, Luzhou, China; ^2^Model Animal and Human Disease Research of Luzhou Key Laboratory, Southwest Medical University, Luzhou, China; ^3^Gastrointestinal Surgery, Suining First People's Hospital, Suining, China; ^4^Department of Gastroenterology, The Affiliated Hospital of Southwest Medical University, Luzhou, China; ^5^College of Veterinary Medicine, Sichuan Agricultural University, Chengdu, China

**Keywords:** gut microbiota, gut metabolites, male reproductive function, sperm quality, testis structure, sex hormone, sexual behavior, probiotics

## Abstract

Globally, ~8%−12% of couples confront infertility issues, male-related issues being accountable for 50%. This review focuses on the influence of gut microbiota and their metabolites on the male reproductive system from five perspectives: sperm quality, testicular structure, sex hormones, sexual behavior, and probiotic supplementation. To improve sperm quality, gut microbiota can secrete metabolites by themselves or regulate host metabolites. Endotoxemia is a key factor in testicular structure damage that causes orchitis and disrupts the blood-testis barrier (BTB). In addition, the gut microbiota can regulate sex hormone levels by participating in the synthesis of sex hormone-related enzymes directly and participating in the enterohepatic circulation of sex hormones, and affect the hypothalamic-pituitary-testis (HPT) axis. They can also activate areas of the brain that control sexual arousal and behavior through metabolites. Probiotic supplementation can improve male reproductive function. Therefore, the gut microbiota may affect male reproductive function and behavior; however, further research is needed to better understand the mechanisms underlying microbiota-mediated male infertility.

## 1 Introduction

It is estimated that about 8%−12% of couples worldwide have fertility problems, of which about 50% are contributed by men (Agarwal et al., 2021). Apart from idiopathic and unclassified infertility, the proportion attributed to male infertility accounts for 20% in Western countries (de Kretser, 1997) and 18.8% in oriental countries (Zheng et al., 2019). The increased incidence of male infertility is thought to be related to exogenous factors (Al-Asmakh et al., 2014). The gastrointestinal tract mediates not only the efficient uptake of nutrients but also the absorption of exogenous factors. Gut microbiota is an important part of the gastrointestinal tract, regarded as an extensive “endocrine organ” of the host (Ly et al., 2021), the importance of the gut microbiota has been increasingly recognized in the past few years.

The gut microbiota in humans mainly include Firmicutes, Bacteroides, Proteus, Actinomycetes, Fusobacteria and Verrucomicrobia (Consortium, 2012), and they possess over 3 million genes while the human genome contains only approximately 23,000 genes, playing an important role in host physiology (Lloyd-Price et al., 2016). For example, proteins and peptides that escape host digestion can be converted into a variety of bioactive compounds by the gut microbiota (Liu et al., 2022). These metabolites are classified into three types: (1) metabolites directly produced from the diet, such as short-chain fatty acids (SCFAs), polyunsaturated fatty acids (PUFAs), and amino acid derivatives; (2) de novo metabolites, such as lipopolysaccharide (LPS) and K vitamins; and (3) metabolites produced by the host and modified by gut microbiota, such as secondary bile acids and hydroxysteroid dehydrogenase (HSDH) (Liu et al., 2022). These metabolites are crucial for regulating host metabolism, not only in regulation of immunological, metabolic and neurological function (Adak and Khan, 2019), but also in male reproductive function.

The impact of gut microbiota on male reproductive function is a double-edged sword. On one hand, gut microbiota can improve spermatogenesis and sperm motility, and treat male infertility. For example, alginate oligosaccharides (AOS) have been used to improve semen quality and testicular microenvironments through modulating gut microbiota caused by HFD and T1DM (Zhao et al., 2020; Hao et al., 2022a,b). Taxifolin also improved semen quality by improving gut microbes and blood metabolites in Duroc boars (Zhou et al., 2022). *Lactobacillus, Bifidobacterium*, and *Enterococcus* can enhance sperm quality by alleviating inflammatory response (Zhang et al., 2023). One the other hand, gut dysbiosis can also disrupt reproductive function. Gut microbiota and its metabolites can activate abnormal immune signals via LPS, bind to TLR-24 complexes, and increase the production of proinflammatory cytokines and ROS/RNS (Wiest and Garcia-Tsao, 2005), and are involved in inflammation-induced damage to testicular structures (Palladino et al., 2018; Tremellen et al., 2018). For example, mucolytic bacteria such as *Bacteroides caccae* and *Akkermansia muciniphila* will increase the activity of mucin-degrading enzymes and use the mucus glycoproteins secreted by the host as a source of nutrition, leading to the erosion of the colonic mucus barrier (Desai et al., 2016). Structural damage to the intestinal barrier leads to increased translocation of LPS and reduced production of SCFAs by the intestinal microbiota (Li et al., 2022c), leading to low-grade inflammation, metabolic disorders, endocrine disorders, and insulin resistance, all of which affect spermatogenesis (Wang and Xie, 2022). *Prevotella copri* has also been suggested as a possible important cause of spermatogenic defects (Ding et al., 2020). These findings contribute to the probability of the existence of the gut microbiota-testis axis.

This review focuses on the relationship between gut microbiota and the male reproductive system to gain a comprehensive understanding of why gut microbiota is related to male infertility. We hope that this review will serve as a modest spur to encourage more scholars to come forward with their valuable contributions, inspire them to explore how it is related, and provide fresh insights into the diagnosis and treatment of male reproductive dysfunction in clinical practice.

## 2 Gut microbiota change sperm quality

The gut microbiota is involved in a variety of male reproductive physiological processes and can affect sperm quality. For example, studies have found that sperm motility in Duroc pigs can be improved by the gut microbiota, especially *Rikenellaceae* (Li et al., 2022b). Probiotic supplements are effective in repairing spermatogenic impairment (Zhang et al., 2023). Most of these effects are mediated by metabolites produced by the gut microbiota. In this section, we will refer to some types of typical metabolites to argue for this, especially for accelerating Ca^2+^ influx to sperm, stabilizing the sperm membrane, maintaining sperm energy supply, and participating in spermatogenesis.

### 2.1 Metabolites of gut microbiota improve sperm motility

Tryptophan is an essential amino acid that plays a vital role in the daily diet. There are three primary metabolic pathways for tryptophan in the gastrointestinal tract: indole derivatives, kynurenine pathway, and 5-hydroxytryptamine (5-HT) pathway. Among these pathways, the kynurenine pathway is regulated by the major rate-limiting enzyme Indoleamine2,3-dioxygenase1 (IDO1), whose synthesis can be expedited by the gut microbiota. In the 5-HT pathway, tryptophan hydroxylase 1 (TpH1) is the key enzyme responsible for 5-HT synthesis by colonic enterochromaffin cells (ECs) (Agus et al., 2018). Gut microbiota is of great significance in this process, specifically for indigenous spore-forming bacteria (Yano et al., 2015). For example, *Clostridium ramosum* (Mandić et al., 2019) and *Blautia* (Golubeva et al., 2017) were proved to promote 5-HT secretion from ECs. This specific mechanism may be related to the increased expression levels of Tph1, Sert, and Ido1. Tph activity determines the amount of 5-HT produced and released, whereas Sert controls the rate of 5-HT reuptake and breakdown (Mawe and Hoffman, 2013). Although the precise mechanism remains unclear, it is certain that the gut microbiota has a close affinity for tryptophan metabolism and the 5-HT pathway, which may be due to its metabolite SCFAs (Reigstad et al., 2015).

Furthermore, 5-HT exists in the sperm and is involved in sperm physiology. It can be observed in the mid-segment of human sperm (Jiménez-Trejo et al., 2012). A similar pattern for 5-HT selective reuptake transporter (SERT) was found in horse sperm (Gao et al., 2018). 5-HT can activate transmembrane adenylate cyclase (tmAC) and open membrane Ca^2+^ channels (CatSper), resulting in an influx of Ca^2+^ and the activation of protein kinase A (PKA) (Alasmari et al., 2013), which is involved in regulating tyrosine phosphorylation and sperm protein Ser/Thr phosphorylation (Kwon et al., 2014; Bae et al., 2020; Kushawaha et al., 2020). In addition, related to both CatSper and Ca^2+^ stores, the spermatozoon is hyperactivated, which is essential for sperm motility and fertilization (Alasmari et al., 2013). This range of Ca^2+^ influx can lead to improved sperm quality.

**Figure 1:**
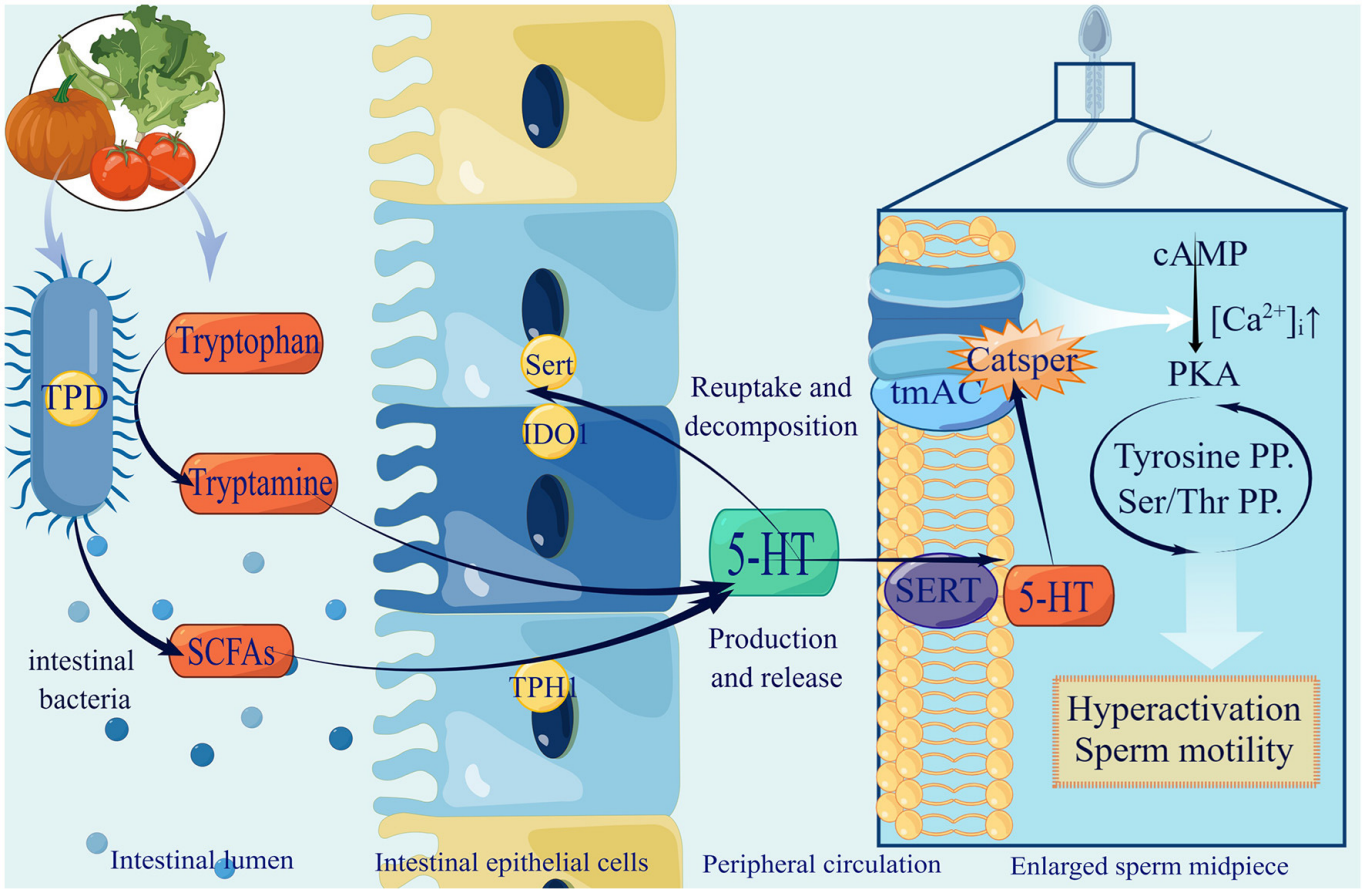
The production of gut microbiota-derived 5-HT and utilization in spermatozoa. (1) Gut microbiota improve tryptamine by expressing tryptophan decarboxylase (TPD), and secrete SCFAs to promote 5-HT by stimulating ECs expressing tryptophan hydroxylase (TPH1), selective reuptake transporter (SERT) and Indoleamine2,3-dioxygenase1 (IDO1). After serum 5-HT passes testis through blood circulation and enters sperm membrane through SERT, it activates tmAC and opens CatSper, resulting in Ca2+ influx increasingly, activating PKA, ultimately enhancing sperm hyperactivation and promoting sperm motility. (2) 5-HT in green suggests that recent research has only shown elevated levels in the central nervous system. It remains to be confirmed whether the gut microbiota can raise the levels of 5-HT in the testes.

Although it has been proven that gut microbiota can improve tryptophan in serum and 5-HT in the central nervous system (Clarke et al., 2013), studies on whether 5-HT secreted with the help of gut microbiota can enter the testis through the blood circulation and play a role directly have yet to be conducted. Therefore, we supposed 5-HT secreted by the synergy of ECs and gut microbiota as a key factor that can enter the testis via blood circulation and maintain an effective concentration, which affects sperm motility partly by regulating Ca^2+^ (Figure 1).

### 2.2 Metabolites of gut microbiota stabilize sperm membrane and maintain sperm motility

Unsaturated fatty acids (UFAs) are fatty acids with double bonds in the hydrocarbon chains. They can be divided into monounsaturated fatty acids (MUFAs) and PUFAs. MUFAs do not have the potential adverse effects of PUFAs, such as lipid peroxidation, and can be used as precursors for PUFAs synthesis. PUFAs, especially long-chain polyunsaturated fatty acids, such as α-linoleic acid (ALA), arachidonic acid (ARA), and docosahexaenoic acid (DHA), are considered as essential fatty acids in the human body. Studies have found that PUFAs have important biological effects: (1) anti-inflammatory effects by interfering with the TLR4/MyD88 pathway and the GPR120/NF-κB pathway; (2) anti-oxidation effects by increasing the expression and activity of catalase and superoxide dismutase (SOD) to reduce reactive oxygen species (ROS) (Korbecki et al., 2019); and (3) increased stability and fluidity of lipid rafts (Kotlyarov and Kotlyarova, 2021) to ensure sperm activity (Castellini et al., 2022).

Interestingly, the gut microbiota can change the content and proportion of PUFAs in the body (Albouery et al., 2019). One of the reasons for this is the biotransformation by microorganisms. For example, *Bacillus proteus* and *Lactobacillus plantarum* reduce gut PUFAs levels by converting linoleic acid (LA) and ALA into stearic acid (Blanchard et al., 2013). Another reason may be that intestinal bacteria can regulate lipid droplet accumulation (Danielli et al., 2023) to affect the absorption of PUFAs.

For male reproductive function, PUFAs mainly affect sperm membrane stability, sperm motility, acrosome reaction, and sex hormone synthesis and increase the anti-inflammatory and antioxidant capacity of testicular cells (Please see Table 1 for details.).

**Table 1:** Effects of unsaturated fatty acids from gut microbiota on male reproductive function.

**UFAs**	**Effects in male reproduction**	**Related gut microbiota**
**MUPAs**		\
OA		*Escherichia coli* (Ma et al., 2022b), *Lachnospiraceae* (Vascellari et al., 2020)
**Omega-3 PUFAs**	Reduce endotoxemia, inflammation and oxidation damage (Kaliannan et al., 2015; Sundaram et al., 2020; Ferramosca and Zara, 2022); change fatty acid composition of the sperm membrane (Castellini et al., 2022) and are closely associated with sperm motility (Rodríguez et al., 2019).	\
ALA	As the precursor of DHA, DPA and EPA (Salem et al., 2001; Rodríguez et al., 2019).	*Bifidobacterium* (Fu et al., 2021)
DHA	Enriched in the head of human sperm (Esmaeili et al., 2015), DHA stabilizes sperm membrane (Castellini et al., 2022); restores serum testosterone levels and future sperm quality in juvenile male rats (Menezes-Garcia et al., 2020); maintains sperm quality (Shishikura et al., 2019) and improves sperm structure and function (Moallem et al., 2015); improves insulin resistance (Zhuang et al., 2021); ameliorates the damage in the testicular microenvironment caused by HFD and DM to save spermatogenesis and improve semen quality (Hao et al., 2022b; Yan et al., 2022). DHA deletion resulted in a shrunken testis, oligospermia, and spermatogenic epithelium disorder (Roqueta-Rivera et al., 2010; Hale et al., 2019) and increased spontaneous acrosome reaction (Bunay et al., 2021).	*Prevotella_9* and *Enterococcus* (Geng et al., 2021), *Bifidobacterium* (Fu et al., 2021)
DPA	Converted into DHA for functioning (Moallem et al., 2015), maintain sperm quality (Shishikura et al., 2019) and improve sperm structure and function (Moallem et al., 2015); negative correlation with acrosome reaction (Lee et al., 2020).	Shigella.spp (Xiao and Kashyap, 2022), *Bifidobacterium* (Fu et al., 2021)
EPA	Improves insulin resistance (Zhuang et al., 2021), ameliorates the impaired testicular microenvironment caused by HFD and DM in order to rescue spermatogenesis and semen quality (Hao et al., 2022b; Yan et al., 2022).	*Bifidobacterium* (Fu et al., 2021)
**Omega-6 PUFAs**	Omega-6 PUFAs in Leydig cells facilitate male sex hormone production (Ri et al., 2022).	\
LA	Ensures sperm membrane integrity and sperm motility (Mourvaki et al., 2010); activates GPR120/ERK signaling pathway in Leydig cells and upregulates 3β-HSDH and StAR to promote testosterone (Xu et al., 2020); confers host resistance to HFD-induced obesity and inflammation (Miyamoto et al., 2019).	*Bacillus proteus, Lactobacillus plantarum* converts LA to stearic acid (Blanchard et al., 2013), HYA, and ARA (Miyamoto et al., 2019).
ARA	Acts as a lower substitute for DHA (Roqueta-Rivera et al., 2010); promotes intracellular [Ca^2+^] increase in round sperm (Paillamanque et al., 2016).	

OA, oleic acid; ROS, reactive oxygen species; PUFAs, polyunsaturated fatty acids; ALA, α-Linolenic acid; DHA, docosahexaenoic acid; DPA, docosapentaenoic acid; EPA, eicosapentaenoic acid; LA, linoleic acid; HFD, high fat diet; DM, diabetes mellitus; ERK, extracellular regulated protein kinases; GPR120, G Protein Coupled Receptor 120; HSDH, hydroxysteroid dehydrogenase; StAR, steroidogenic acute regulatory protein; HYA, 10-hydroxy-cis-12-octadecenoic acid; ARA, arachidonic acid.

### 2.3 Metabolites of gut microbiota promote sperm energy supply

Testicular tissue is a hypoxic organ that primarily uses the lactate produced by glycolysis within Sertoli cells as an energy substrate. Glucose transporters (GLUT) in Sertoli cells transport glucose from the interstitial fluid of seminiferous tubules to the cytoplasm of Sertoli cells for glycolysis. The resulting lactate is transported out of the cells through the monocarboxylate transporter (MCT) 4 on Sertoli cells, and then transported into spermatogenic cells by MCT2 for energy supply (Rato et al., 2012). Even under aerobic conditions, the head and principal piece (tail) of the spermatozoon are capacitated mainly by glycolysis (du Plessis et al., 2015).

Gut microbiota and its metabolites can promote glycolysis to maintain sperm energy supply and enhance sperm motility. For example, leucine supplementation can improve the average curve speed of sperm in boars (Lin et al., 2022). 5-HT can keep tryptophan away from the melatonin pathway through the kynurenine pathway to reduce circulating melatonin levels (Laborda-Illanes et al., 2021), which play a vital role in male reproduction, including promoting the production of lactate by Sertoli cells, activating some glycolysis-related enzymes (Rocha et al., 2014), and increasing the energy supply of sperm (Rato et al., 2012). In addition, *Bacteroides, Streptococcaceae, Akkermansia* are thought to improve the energy supply for spermatid cells by improving glycolysis (Zhu et al., 2021). Previous studies have found that some drugs, such as Pioglitazone (Meneses et al., 2016), Metformin (Alves et al., 2014), Spermidine (Wang et al., 2022) and Nicotinamide mononucleotide (Ma et al., 2022a), can support spermatogenesis by benefitting gut microbiota and improving the glycolysis of Sertoli cells.

In addition, the gut microbiota can regulate blood glucose levels, which is also an important substrate for supporting testicular glycolysis. For example, bile salt hydrolase (BSH) producing bacteria, Barnesiella and Clostridium XlVa, promote the production of unconjugated cholic acid (CA), chenodeoxycholic acid (CDCA), and the secondary bile acid deoxycholic acid (DCA) to inhibit hepatic gluconeogenesis via the FXR-SHP-FOXO1 pathway, thereby reducing blood glucose levels (Zhuang et al., 2021). And in one study, metformin was used to alter folate and methionine metabolism to inhibit the growth of B. fragilis, reduce BSH activity, thereby inhibiting intestinal FXR signaling (Sun et al., 2018) which can induce endoplasmic reticulum (ER) oxidative stress to significantly attenuate mitochondrial citrate synthase activity, triggering an increase in hepatic mitochondrial acetyl-CoA levels and pyruvic carboxylase (PC) activity (Xie et al., 2017). Furthermore, September Numata et al. also found a molecular basis to support our argument: not only SCs express GLUT, but sperm can also express a Na+-dependent sodium-glucose cotransporter (SGLT), whose deletion decreases glucose uptake, glycolytic activity, and ATP production (Numata et al., 2022). Therefore, we hypothesized that gut microbiota may alter sperm motility by regulating blood glucose levels through metabolites that affect SCs and sperm glycolysis. All in all, gut microbiota and its metabolites may act on testicular glycolysis and regulate blood glucose to affect germ cell energy supply and altering sperm motility. Metabolites of Gut Microbiota Participate in Spermatogenesis.

Insulin-like growth factor type I (IGF-1) is a key factor in maintaining the pluripotency of mouse spermatogonial stem cells (Huang et al., 2009). Gut microbiota metabolites such as branched chain amino acids (BCAAs) can also improve the secretion of IGF-1 (Pedrosa et al., 2013; Habibi et al., 2022). Serum IGF-1 levels can be increased by supplementation with *Bacillus amyloliquefaciens C-1* and *Bacillus subtilis* (Du et al., 2018). IGF-1 can activate the Ras/MAPK, Ras/ERK (Shen et al., 2014) and PI3K/AKT (Pitetti et al., 2013) pathway to promote cell proliferation, cell differentiation, and cell survival, which can accelerate the differentiation of spermatogonia to primary spermatocytes (Hakuno and Takahashi, 2018; Józefiak et al., 2021). The insulin/IGF signaling pathway are involved in follicle-stimulating hormone-mediated (FSH-mediated) proliferation of immature Sertoli and Leydig cells. Without this, the proliferation and development speed of Sertoli and Leydig cells would slow, leading to testicular shrinkage and reduced sperm production (Pitetti et al., 2013; Neirijnck et al., 2018).

## 3 Gut microbiota disrupt the testicular structure

Using fecal microbiota transplantation (FMT) from HFD mice, the researchers observed that the recipient mice were infiltrated by T cells and macrophages in the gut, and had a significant increase of pro-inflammatory cytokines in the epididymis and a remarkable decrease in spermatogenesis and sperm motility. This evidence demonstrates an intimate link between microbiota dysbiosis and male infertility (Ding et al., 2020). Furthermore, Zheng et al. directly observed that macrophages and dendritic cells capture spermatozoa in the caudal cavity of the epididymis for the first time, directly proving that chronic epididymitis is a possible cause of oligospermia in patients (Zheng et al., 2021). We suggest that one of the potential reasons for the association between gut microbiota and male infertility is elevated LPS, which can result in local testicular inflammation and oxidative stress disorders, and does harm to the normal testicular structure such as the BTB (Shen et al., 2022).

### 3.1 Gut microbiota cause endotoxemia and orchitis

Endotoxemia refers to systemic inflammation caused by a poorly regulated host response to LPS (Płóciennikowska et al., 2015). When intestinal barrier permeability changes, microbial-related molecular patterns (MAMPs), such as LPS, lipoproteins, peptidoglycans, and lipoproteins, can cross the intestinal barrier and enter the circulation, then arrive at the testicles through the testicular artery and bind to pattern recognition receptors (PRRs) on the cell membrane of testicular cells, eventually causing cellular oxidative stress, local inflammation and destruction of the testicular structure (Wiest and Garcia-Tsao, 2005). The most typical and main-affecting MAMPs are LPS. The entry of LPS into circulation is believed to be a frequent cause of idiopathic male infertility due to systemic inflammation and oxidative stress (Agarwal et al., 2017).

Studies suggest that the classical inflammatory pathway, the Toll-like receptor (TLR) pathway, is involved in the response to orchitis induced by endotoxemia (Palladino et al., 2018). The TLR4/MyD88 pathway can activate IKK to induce orchitis via NF-kB (Saad et al., 2016), elevate the level of inflammation and oxidative stress, and finally result in direct damage to Leydig cell function and sperm DNA damage (Pearce et al., 2019). In addition, LPS-mediated TLRs activation can inhibit MAPK, p38, ERK, and JNK signaling, leading to autophagic dysfunction (Wang et al., 2021). This disrupts the ability of testicular cells to resist inflammation and oxidative stress, which leads to testicular injury in orchitis.

### 3.2 Gut microbiota damage BTB

BTB is formed by the cellular junction of adjacent Sertoli cells at the base of the seminiferous tubule. It is composed of capillary endothelium, basement membrane, connective tissue, and multiple types of cell junctions from Sertoli cells, mainly consisting of tight junctions, basic electrophysiological specializations, gap junctions, and cell-like junctions (Neto et al., 2016; Lustig et al., 2020). Regulators of cell connections in the testis include hormones [such as testosterone (Yan et al., 2008) and androgens (Xia et al., 2005)], cell factors [IL-1a (Sarkar et al., 2008) and TNF-α (Li et al., 2006; Xia et al., 2009)], growth factors [HGF (Catizone et al., 2012), NO (Lee and Cheng, 2008) and TGF-β3 (Xia et al., 2009)], and gut microbiota.

**Figure 2:**
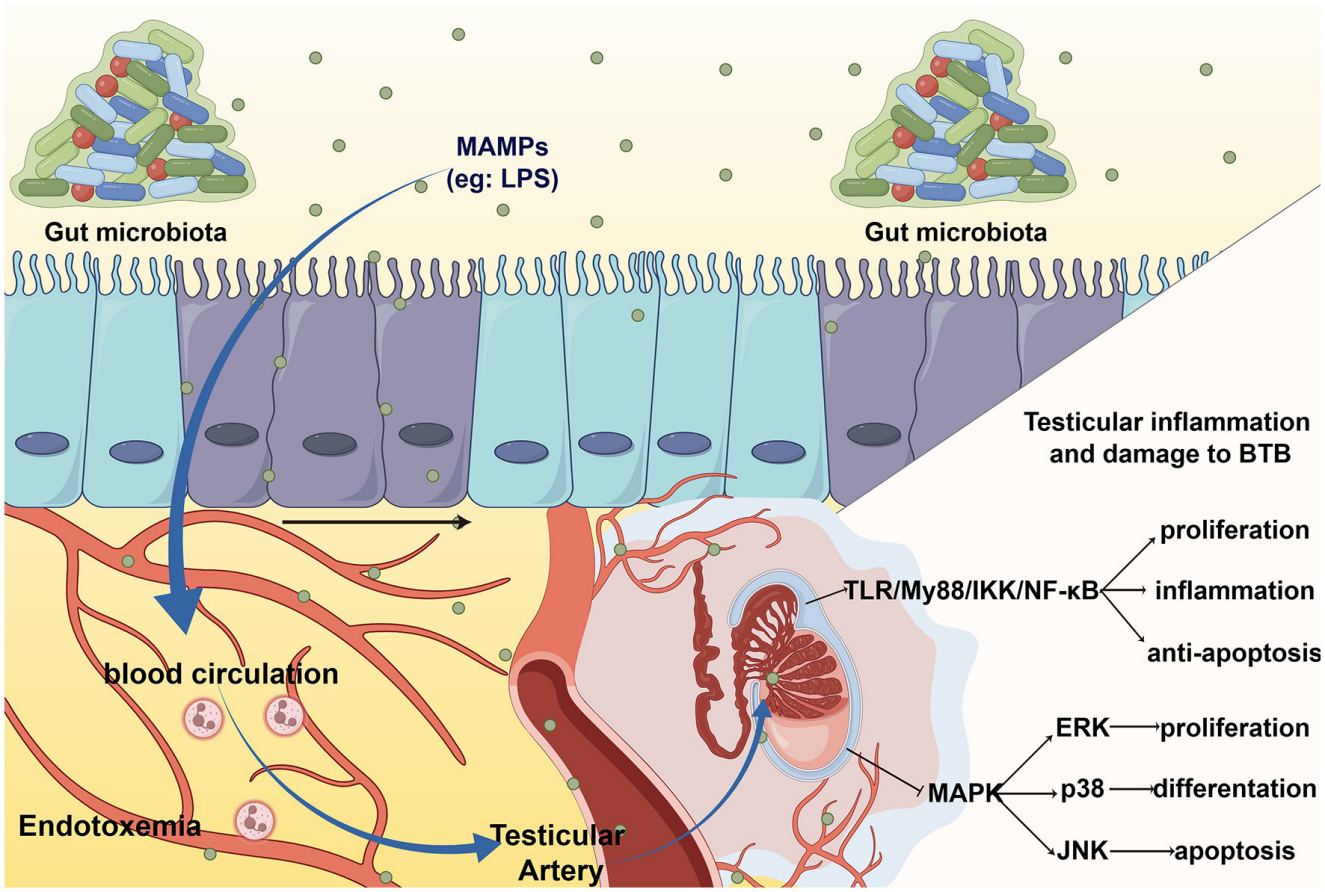
The entering of MAMPs to testis leads to testicular inflammation and BTB damage. MAMPs, such LPS, enter the blood circulation through intestinal barrier and cause systemic endotoxin. When LPS arrive at the testes via the testicular artery, it activates toll-like receptor (TLR) cause orchitis and damage to the BTB.

The detrimental impact of the gut microbiota on BTB can be partially ascribed to inflammation and oxidation, mainly caused by LPS. A study found that LPS treatment can lead to orchitis and significantly decrease the expression of cell junctions, such as testicular intercellular adhesion molecule 1, tight junction protein 1, and gap junction α-1 protein (Shen et al., 2022). Furthermore, IL-6, induced by gut microbiota, can damage the tight junctions of Sertoli cells by disrupting the ERK-MAPK signaling pathway and changing the localization and number of BTB component proteins (Zhang et al., 2014). This change in BTB permeability can be restored to the levels of cell adhesion proteins by supplementation *Clostridium butyricum*, a high-level butyric acid secretor (Al-Asmakh et al., 2014). In summary, elevated LPS and systemic endotoxemia can damage the normal structure of the BTB by causing inflammatory damage and disrupting cell junctions (Figure 2).

## 4 Gut microbiota regulate sex hormones

There is cross-talk between the gut microbiota and endocrine system. Researchers have found that gut bacteria are highly correlated with sex hormones, especially testosterone, in various subjects. For example, in pubertal subjects, the abundances of *Adlercreutzia, Ruminococcus, Dorea, Clostridium*, and *Parabacteroides* were significantly correlated with testosterone levels (Yuan et al., 2020). In men, the abundance of *Acinetobacter, Dorea, Ruminococcus, Megamonas*, and *Lactobacillus* was also notably related to testosterone levels (Shin et al., 2019; Akram et al., 2022). In germ-free (GF) mice, serum interstitial cell stimulating hormone (ICSH), follicle-stimulating hormone (FSH), and testosterone levels in the testes were decreased (Al-Asmakh et al., 2014). We attempted to explain this phenomenon from three aspects: the gut microbiota can affect the HPT axis, modify hydroxysteroid dehydrogenase (HSDH), and regulate the enterohepatic circulation of sex hormones.

### 4.1 Gut microbiota affect HPT axis

The HPT axis means that the hypothalamus releases gonadotrophin releasing hormone (GnRH) to promote pituitary release ICSH and FSH, and the two can respectively regulate the release of testosterone from Leydig cells and anti-Mullerian (AMH) from Sertoli cells in the testis, also there exists a corresponding negative feedback mechanism (Aleksic et al., 2022). The gut microbiota can participate in the regulation of gonadal development and affect each level of the HPT axis (Shen et al., 2022). This includes both the supportive role of probiotics and damaging role of pathogenic bacteria.

Probiotics can restore damage to the HPT axis. Gut microbiota can improve insulin-like growth factor receptor type I (IGF1R), possibly through SCFAs (Yan and Charles, 2018). Supplementation with *Bacillus amyloliquefaciens C-1* and *Bacillus subtilis* increased the abundance of SCFA-producing bacteria and elevated serum IGF-1 concentrations (Du et al., 2018). IGF-1 appears to be involved in the proliferation and differentiation of Sertoli cells by mediating the effects of FSH through the PI3K/AKT pathway (Cannarella et al., 2018). Moreover, IGF-1 can participate in mitochondriogenesis in Leydig cells and affect steroid hormone synthesis (Radovic et al., 2019). In a mouse model lacking the insulin receptor (InsR) and IGF1R, the adrenal cortex and testis are significantly shrunk along with develop-inhibited Leydig cells, leading to low corticosterone and testosterone (Neirijnck et al., 2018).

However, the entry of LPS into circulation is hypothesized to be a key factor in triggering male hypogonadism (Tremellen, 2016). LPS, which is mainly produced by cytoderm lysis of gram-negative bacteria, can act on all levels of the HPT axis independently and eventually damages the male gonad. LPS treatment resulted in the activation of the medial preoptic area (mPOA) of the hypothalamus, increased FSH and ICSH release, and HPT axis disorder (Shen et al., 2022). Studies have shown that elevated LPS may directly result in compromised Leydig cells functionality (Tremellen et al., 2018). Even low doses of LPS can drive the body's inflammatory response independently of FSH and ICSH, damage Leydig cells, and lower serum testosterone (Tremellen et al., 2017, 2018). However, after 6 h of low-dose LPS injection, treatment with RAPID (reduced temperature, acidification, protease inhibition, isotope exogenous control, and dilution) effectively increased corticotropin-releasing hormone (CRH) and adrenocorticotropic hormone (ACTH) levels (Goebel et al., 2011). This provides a methodological basis and valuable opportunity for us to deal with acute endotoxemia and save male reproductive function.

### 4.2 Gut microbiota regulate sex hormones by HSDH

Ever since the first evidence of cortisol synthesis by microbiota was found (Nabarro et al., 1957), the human gut microbiome (HGM) has gradually been recognized to play a more important role in steroid modification than the host, which is critical for estrous cycles, testosterone levels, and reproductive function (Cross et al., 2018).

Most bacteria located in the intestinal and urogenital tracts encode cortisol-inducible operons. These include steroid-17,20-desmolase, synthesized by HGM, which can inactivate cortisol by cutting the side chain (Devendran et al., 2018). Typical representatives, such as *Clostridium scindens* and *Propionimicrobium lymphophilum*, have extremely high activities against glucocorticoids (Ly et al., 2020).

Numerous studies have shown that the gut microbiota can produce HSDH and impact male reproductive health. Here, we list some common examples: *Mycobacterium neoaurum* can degrade serum testosterone with the help of 3β-HSDH (Li et al., 2022a). Compared to wild-type *Comamonas testosterone*, the 7α-HSDH knockout mutant showed reduced degradation of testosterone, estradiol, and cholesterol (Ji et al., 2014). As an important glucocorticoid metabolizing enzyme in Leydig cells, 11β-HSDH is involved in regulating steroidogenic gene expression and testosterone production in Leydig cells (Wang et al., 2019). *Butyricicoccus desmolans* and *Clostridium cadaveris* have previously been reported to demonstrate steroid-17,20-desmolase and 20β-HSD activities, which are responsible for the formation of androstanes from cortisol (Devendran et al., 2017).

Notably, the conversion of dehydroepiandrosterone, androstenedione, and testosterone to dihydrotestosterone (DHT) is an essential function of 17β-HSDH enzymes (Bélanger et al., 2003). Some attempts have been made to introduce gene fragments of 17β-HSDH producing bacteria into *Mycobacterium smegmatis* by genetic engineering technology, and natural sterols have been bioconverted to produce testosterone successfully (Fernández-Cabezón et al., 2017). In human patients, 17β-HSDH deficiency caused by related gene mutations also results in low serum testosterone (Ben Rhouma et al., 2017; Yang et al., 2017). Perhaps the usage of the bacterial-derived HSDH production pathway will become a new dawn for patients with HSDH-related diseases, but whether it has the same effects as endogenous HSDH remains to be studied. Although the role of microbial HSDH has not been fully elucidated, it has therapeutic potential as a steroid pool modulator or drug targets in the future (Doden and Ridlon, 2021).

Overall, the expression of HSDH by gut microbiota forms the physiological basis for intestinal bacteria to affect the host's sex hormones.

### 4.3 Gut microbiota are involved in the enterohepatic recirculation of sex hormones

In general, active hormones bind to glucuronides in the liver to produce the main metabolites of testosterone (T), such as testosterone glucuronide (TG), androsterone glucuronide (AG), etiocholanolone glucuronide (EtioG), and dihydrotestosterone glucuronide (DHTG). These metabolites are transported via the bile duct, with one part entering the blood and the other part excreted through urine and feces (Auer et al., 2020). Notably, intestinal microorganisms can mediate the deglucuronidation of DHT and T during this process, releasing inactive free androgens for host re-absorption (Colldén et al., 2019). This may be related to *Clostridia*, which codes for β-glucuronidase and participates in the deglucuronidation of DHT and T (Gloux et al., 2011; Flores et al., 2012; Colldén et al., 2019). However, there is a lack of adequate research to prove that sex hormones deglucuronidated by gut microbiota can affect male reproductive function or sexual behavior, and further research efforts still need to be invested in order to explore this promising issue in greater depth.

## 5 Gut microbiota influence sexual behavior

Neural networks play a crucial role in mediating various aspects of male sexual behavior. The regions of the brain responsible for regulating male sexual arousal and performance comprise the amygdala, bed nucleus of the stria terminalis (BNST), medial preoptic area (mPOA), paraventricular nucleus (PVN), and mesolimbic dopamine (DA) system (Hull and Dominguez, 2019).

The gut microbiota also has a regulatory effect on the central nervous system to change sexual behavior. LPS produced by gut microbiota can lead to abnormal activation of mPOA (Shen et al., 2022), which affects sexual arousal and ejaculation and is crucial for the completion of sexual behavior (Everitt, 1990). Gut microbiota can promote glucagon-like peptide 1 (GLP-1) secretion (Koopen et al., 2022), and the activation of GLP-1R, particularly within the nucleus of the solitary tract, has been shown to inhibit sexual behavior in sexually immature male mice (Vestlund and Jerlhag, 2020). Moreover, the gut microbiota is also an important factor in maintaining proopiomelanocortin levels (Vagnerová et al., 2019), the precursor of ACTH, which increases the production of endothelin in the adrenal fascicular zone and is associated with male erectile activity (Hadley and Haskell-Luevano, 1999).

Moreover, the dopaminergic system is crucial in the initial phases of sexual behavior, including sexual arousal, motivation, and reward (Melis et al., 2022). Recent studies have shown that gut microbiota, such as *Bacillus spp., Escherichia coli, Proteus vulgaris, Serratia marcescens, Staphylococcus aureus, Hafnia alvei, Klebsiella pneumoniae*, and *Morganella morganii* (Strandwitz, 2018), are involved in both the synthesis and degradation of intestinal phenylalanine and DA. Especially *Bifidobacterium* can increase intestinal phenylalanine, a DA precursor, via cyclohexadienyl dehydratase (Aarts et al., 2017). A study has found that *Lactobacillus plantarum* have been shown to improve sexual behavior via improving DA activity and preventing DA loss (Edem et al., 2021).

It is noteworthy that studies to date have only proved the increase of DA precursor and then a higher DA level in the brain rather than in the testis. Therefore, studies on the sexual behavior effect of DA by the gut microbiota mainly focus on the microbiota-gut-brain axis rather than the microbiota-gut-testis axis. In the future, further studies are warranted to explore whether DA produced by the gut microbiota can act directly in the testis. However, it is certain that DA in the gastrointestinal tract is produced by phenylalanine from food digestion (Franco et al., 2021) and gut microbiota can participate in the regulation of sexual behavior by the gut-brain axis or gut-hypothalamic axis.

## 6 Gut microbiota are used for the treatment of male infertility

Therapies targeting the gut microbiome typically include probiotics, prebiotics, and microbiota restoration therapies. Probiotics are agents of microorganisms to improve gut microbial balance (Gibson and Roberfroid, 1995), while prebiotics are non-digestible compounds (e.g., fructooligosaccharides, galacto-oligosaccharides, and xylooligosaccharides) that benefit the host by modulating gut microbiome (Valcheva and Dieleman, 2016). Microbiota therapy includes FMT, symbiotic microbial consortia, or engineered symbiotic microbes (Sorbara and Pamer, 2022), and reconstructs dysregulated microbiota with a healthy microbiota (Alli et al., 2022). Through intervening with the host metabolome in methods above, gut microbiota are linked to the regulation of testicular function and have potential value for the treatment of male infertility (Zhang et al., 2022).

### 6.1 Probiotics

Probiotics are live bacteria that offer a range of potential health benefits to the human body. Oral supplementary probiotics, *Lactobacillus, Bifidobacterium, Enterococcus, Collinsella*, and *Blautia*, can enhance sperm quality by alleviating sperm inflammatory response and oxidative stress (Helli et al., 2022; Cao et al., 2023; Zhang et al., 2023). These may be linked to probiotics can regulate the Nrf2-Keap1-ARE signaling pathway to increase antioxidant activity and enhance neutralization of reactive oxygen species, ultimately resulting in improved sperm concentration and motility in infertile men (Helli et al., 2022).

Above all, it is *Lactobacillus* that are widely believed to have potential benefit for male infertility (Doroftei et al., 2022). We have drawn Table 2 to give a superficial description of recent studies on the effects and mechanisms of lactic acid bacteria on the male reproductive system. Especially *L. rhamnosus* strain NCDC 610 and *L. fermentum* strain NCDC 400 are suggested as alternative options to pharmaceuticals because of their positive impact on weight reduction and their ability to enhance endogenous testosterone levels (Akram et al., 2022). The studies have demonstrated the efficacy of probiotics in addressing male infertility, and their considerable therapeutic potential and extensive development space should not be overlooked. Therefore, we anticipate additional research efforts to delve more deeply.

**Table 2:** The effects and mechanisms of Lactobaciillus on male reproductive function.

**Lactic acid bacteria**	**Studied model**	**Mechanism**	**Effects**	**Related gut microbiota**
Lactic acid bacteria	Rat	Promote SCFAs production; avert gut dysbiosis; antioxidant.	Helps rat testis combat with some toxic substances	(Chen et al., 2022a)
*Lactobacillus reuteri*	Mice	Up-regulate the level of anti-inflammatory factor il-10 and reduce the level of pro-inflammatory factor il-17; have nutrient effect for leydig cell; modulate gastrointestinal immunity and thus exert systemic effects on the immune system, thereby activating metabolic pathways and restoring tissue homeostasis and overall health.	Improve testicular weight and size, leydig cell count, serum testosterone, spermatogenesis, sperm concentration and motility	(Poutahidis et al., 2007, 2014).
*Lactobacillus rhamnosus*	Mice	Antioxidant; lower blood lipids for protecting leydig and sertoli cells	Lose weight; improve testicular weight and size, leydig cell count, sperm motility, and spermatogenesis; repair the hpt axis by enhancing the serum levels of lh, fsh, and testosterone.	(Ooi and Liong, 2010; Pinto-Fochi et al., 2016; Dardmeh et al., 2017)
*Lactobacillus plantarum*	Mice	Improve da activity and prevent da loss	Improve sexual behavior	(Edem et al., 2021).
*Lactobacillus paracasei*	Human	Ameliorate the gut microbiota, improve the prostatic microenvironment, optimize the free radical concentration in the seminal fluid.	Improve the quality and quantity of spermatozoa: volume of the ejaculate, sperm count, sperm concentration, progressive motility, the percentage of typical forms, and their fsh, lh, and testosterone levels.	(Maretti and Cavallini, 2017)

SCFA, short-chain fatty acids; HPT, hypothalamic-pituitary-testis; LH, luteinizing hormone; FSH, follicle-stimulating hormone; DA, dopamine.

### 6.2 FMT

**Figure 3:**
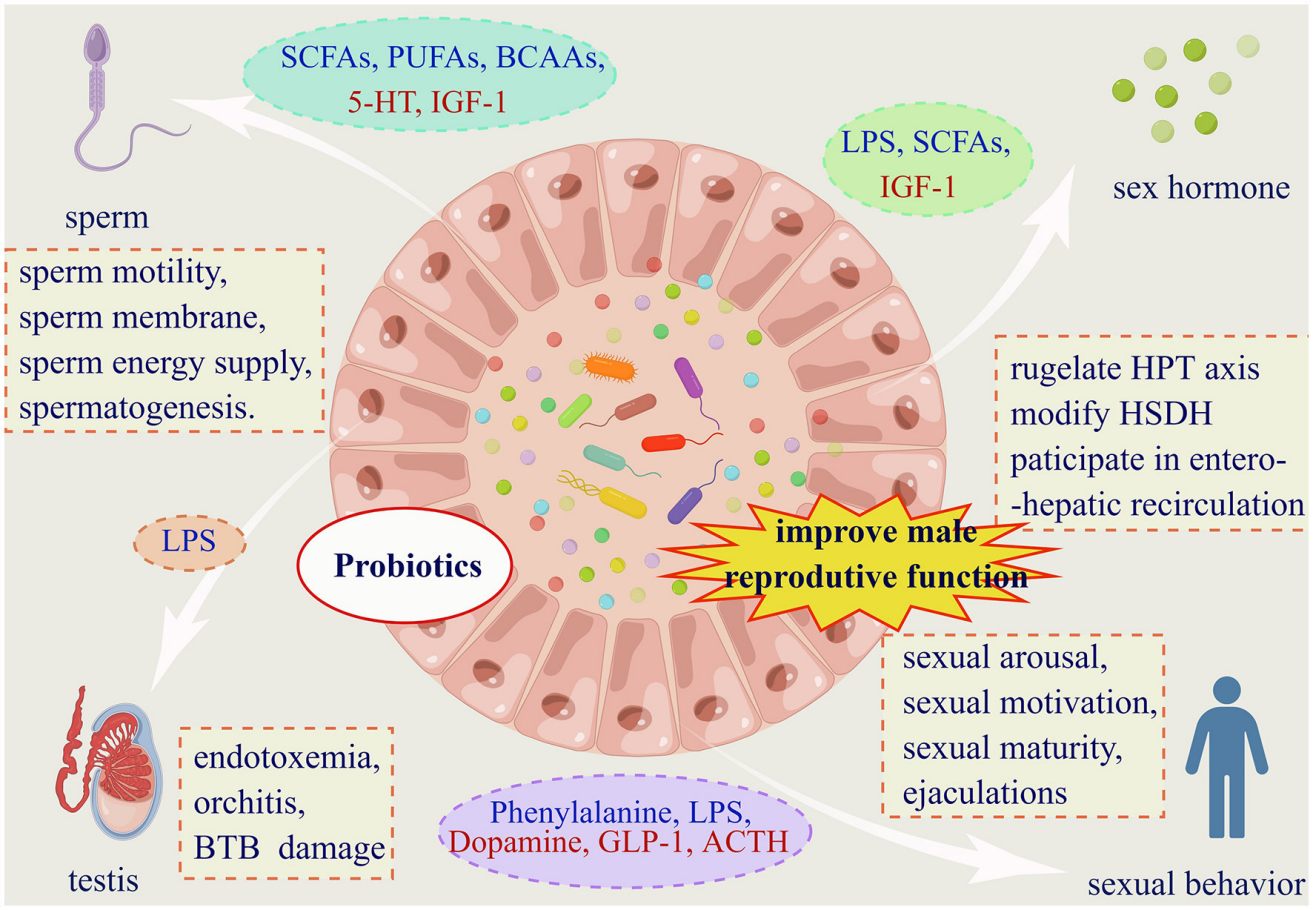
Gut microbiota affects male reproductive function in four dimensions. (1) Gut microbiota affects male fertility via four facets. And probiotics supplementation is proved to have pharmaceuticals value for male infertility. (2) Substances from dashed oval box in blue represent those that can be secreted or indirectly elevated by the gut microbiota, and red are reduced. (3) Abbreviations: SCFAs: short chain fatty acids; PUFAs: polyunsaturated fatty acids; BCAAs: branched chain amino acids; 5-HT: 5-hydroxytryptamine; IGF-1: insulin-like growth factor-1; LPS: lipopolysaccharide; GLP-1: glucagon-like peptide-1; ACTH: adrenocorticotropic hormone. (3) Within the dashed boxes are aspects of the impact of the gut microbiota on male reproductive function. HPT axis: hypothalamic-pituitary-testis axis; HSDH: hydroxysteroid dehydrogenase; BTB: the blood-testes barrier.

FMT is a valuable tool for demonstrating the vital role of gut microbiota in regulating host physiology (Antushevich, 2020), and conducts a potential to improve infertility. It is common to see to increase beneficial bacteria by drugs and transplant them through FMT to improve male infertility (Figure 3). For example, by transplanting fecal microbiota from AOS users (AOS-FMT), Zhang et al. showed that the gut microbiota can be used to improve spermatogenesis and treat busulfan-induced male infertility for the first time (Zhang et al., 2021a,b). And AOS-FMT are also used to treating male infertility induced by HFD (Hao et al., 2022a), T2DM (Yan et al., 2022) and T1DM (Hao et al., 2022b). What's more, FMT, from healthy microbiota benefitted by traditional Chinese medicine such as Guijiajiao (Sheng et al., 2023), Radix Rehmanniae and Cornus Officinalis (Chen et al., 2022b) to male patients with impaired fertility, can improve testicular damage and restore spermatogenesis. Even FMT from ordinary healthy mice was able to exert a lesser degree of beneficial effect on the alleviation of testicular damage (Hao et al., 2022b).

These mechanisms are mostly related to restoring the diversity of gut microbiota, changing the blood metabolome of the host (Yan et al., 2022), inhabiting epididymal inflammation (Sheng et al., 2023), and restoring testicular microenvironment (Yan et al., 2022) to improve spermatogenesis and male infertility. For example, AOS-FMT increased beneficial bacteria such as *Bacteroidales, Lactobacillaceae, Bifidobacteria, Sphingomonadales* and *Campylobacterales*, to improve spermatogenesis and semen quality by increasing DHA and EPA in blood metabolome and testis metabolome (Zhang et al., 2021a; Hao et al., 2022b). *Parabacteroides distasonis* transplantation increased polyamine levels in the testis and cecum, and improved testicular histology, testicular index, testicular testosterone levels, expression of genes involved in spermatogenic events, inflammatory factors, and oxidative stress (Zhao et al., 2021).

All in all, FMT may serve as a novel and promising therapeutic approach to improve semen quality and male fertility via the gut microbiota-testis axis.

## 7 Conclusion and perspective

As the eighth emerging organ, the gut microbiota has a far greater influence on the human body than expected. Current evidence suggests that the gut microbiota may influence male reproductive function and behavior, mainly through metabolites. Nonetheless, our understanding of the mechanisms involved in microbiota-mediated male infertility remains limited. Many key questions remain and some of these questions include the following.

- How deeply do gut metabolites influence male reproduction and its mechanism.
- The pharmacokinetics of gut microbiota metabolites and whether they are able to reach the testes at effective concentrations remain unclear.
- More specific mechanisms for the influence of the gut microbiota on the HPT axis are still needed.
- Whether sex hormones modified by intestinal microbiota can play the same role as endogenous sex hormones after being recycled by the host.
- Does the gut microbiota-produced dopamine come into play directly in the sperm rather than through the gut-brain axis?
- More probiotics for male infertility and their pharmacological value are expected to be found.

The methods we currently have for studying the gut microbiota include but not limited to using gnotobiotic animal models, FMT, antibiotic interference, and other approaches, in combination with multi-omics analysis. Among these, manipulating gut microbiota, such as scientific diet, supplementation of probiotics or prebiotics, FMT, and avoidance of antibiotic abuse, is regarded as a more “natural” therapy for working harmoniously with our own natural regulatory systems, which is more widely accepted by patients. Therefore, another research orientation we suggest scholars to study is to develop a more rational and efficient strategy for manipulating the gut microbiota, or develop a more detailed, refined and mature treatment regimen for male infertility. These studies will enhance our understanding of the intensify and mechanisms of the influence of gut microbiota on male reproductive function, and potentially mitigate the disease and improve human health.

All in all, although the pertinence and possibility of gut microbiota treatment for male infertility exists, more specific treatments are still under investigation. We expect that research on the influence of gut microbiota on male infertility will continue to deepen, to reveal more interactions between gut microbiota and male reproductive function and provide more promising protection for human reproductive health.

## Author contributions

SL: Conceptualization, Data curation, Formal analysis, Funding acquisition, Investigation, Methodology, Project administration, Resources, Software, Supervision, Validation, Visualization, Writing—original draft, Writing—review & editing. JH: Conceptualization, Data curation, Investigation, Resources, Validation, Visualization, Writing—review & editing. YL: Conceptualization, Data curation, Investigation, Software, Validation, Writing—review & editing. YW: Conceptualization, Investigation, Project administration, Resources, Writing—review & editing. BC: Conceptualization, Resources, Writing—review & editing. HQ: Data curation, Resources, Validation, Writing—original draft. HC: Project administration, Resources, Supervision, Writing—review & editing. TY: Data curation, Investigation, Methodology, Software, Visualization, Writing—review & editing. LH: Project administration, Resources, Supervision, Writing—review & editing. BF: Project administration, Resources, Supervision, Writing—review & editing. ZY: Project administration, Resources, Supervision, Writing—review & editing. MZ: Project administration, Resources, Supervision, Writing—review & editing. QY: Methodology, Project administration, Supervision, Writing—review & editing. MH: Investigation, Methodology, Resources, Writing—review & editing. WX: Project administration, Resources, Supervision, Writing—review & editing. XZ: Formal analysis, Funding acquisition, Investigation, Methodology, Project administration, Resources, Supervision, Writing—review & editing. CG: Conceptualization, Formal analysis, Funding acquisition, Investigation, Methodology, Project administration, Resources, Software, Supervision, Writing—original draft, Writing—review & editing. RL: Conceptualization, Formal analysis, Funding acquisition, Investigation, Methodology, Project administration, Resources, Supervision, Writing—original draft, Writing—review & editing.
